# Screening and genome-wide analysis of lignocellulose-degrading bacteria from humic soil

**DOI:** 10.3389/fmicb.2023.1167293

**Published:** 2023-08-11

**Authors:** Tianjiao Zhang, Shuli Wei, Yajie Liu, Chao Cheng, Jie Ma, Linfang Yue, Yanrong Gao, Yuchen Cheng, Yongfeng Ren, Shaofeng Su, Xiaoqing Zhao, Zhanyuan Lu

**Affiliations:** ^1^School of Life Science, Inner Mongolia University, Hohhot, China; ^2^Inner Mongolia Academy of Agriculture and Husbandry Science, Hohhot, China; ^3^Key Laboratory of Black Soil Protection And Utilization (Hohhot), Ministry of Agriculture and Rural Affairs, Hohhot, China; ^4^Inner Mongolia Key Laboratory of Degradation Farmland Ecological Restoration and Pollution Control, Hohhot, China; ^5^School of Life Science, Jining Normal University, Ulanqab, China

**Keywords:** cellulose-degrading bacteria, *Bacillus velezensis*, whole-genome sequencing, comparative genomic analysis, carbohydrate-active enzyme

## Abstract

Crop straw contains huge amounts of exploitable energy, and efficient biomass degradation measures have attracted worldwide attention. Mining strains with high yields of cellulose-degrading enzymes is of great significance for developing clean energy and industrial production of related enzymes. In this study, we reported a high-quality genome sequence of *Bacillus velezensis* SSF6 strain using high-throughput sequencing technology (Illumina PE150 and PacBio) and assessed its lignocellulose degradation potential. The results demonstrated that the genome of *B. velezensis* SSF6 was 3.89 Mb and contained 4,015 genes, of which 2,972, 3,831 and 158 genes were annotated in the COGs (Clusters of Orthologous Groups), KEGG (Kyoto Encyclopedia of Genes and Genomes) and CAZyme (Carbohydrate-Active enZymes) databases, respectively, and contained a large number of genes related to carbohydrate metabolism. Furthermore, *B. velezensis* SSF6 has a high cellulose degradation capacity, with a filter paper assay (FPA) and an exoglucanase activity of 64.48 ± 0.28 and 78.59 ± 0.42 U/mL, respectively. Comparative genomic analysis depicted that *B. velezensis* SSF6 was richer in carbohydrate hydrolase gene. In conclusion, the cellulose-degrading ability of *B. velezensis* SSF6 was revealed by genome sequencing and the determination of cellulase activity, which laid a foundation for further cellulose degradation and bioconversion.

## 1. Introduction

Crop straw is one of the most abundant biological sources on Earth (Menshawy et al., 2022; Yang et al., 2022). At present, owing to the problems of abundant straw varieties and large yields, complex straw composition and structure, and low conversion rate of the straw industry (Marriott et al., 2016), as well as the influence of rough treatment methods such as straw incineration and burial, a large amount of straw resources are wasted and accompanied by serious environmental pollution. he return of straw to the field is a crucial step in the innocuous treatment of straw, which can significantly increase soil nitrogen (Ahmed et al., 2020), phosphorus (Bai et al., 2015), potassium (Ma et al., 2010), and other nutrients, which are advantageous for crop growth and development (Chen et al., 2022b), and improve soil nutrients. Therefore, developing quick and efficient techniques for straw treatment is essential.

Agricultural straw primarily comprises of lignocellulosic biomass (LCB) (Ragauskas et al., 2006). Microbial degradation of lignocellulose is a biological treatment method with ecological benefits, compared to physical and chemical methods. By utilizing a variety of members of the carbohydrate-active enzyme (CAZyme) family in concert, cellulose-degrading microorganisms, which act as an intrinsic driving force for the degradation and transformation of biomass, such as straw, degrade carbohydrates into reducing sugars (Sharma et al., 2016), and at the same time degraded lignocellulose is advantageous for microbial growth (Dar et al., 2018). Enzymatic hydrolysis of lignocellulose is a key strategy for the degradation of cellulose because of its specificity, high conversion rate, and ecological character (Pollegioni et al., 2015; Fatani et al., 2021). The ability to rapidly degrade biomass depends on the successful identification of novel strains that generate cellulases, for example, fungi, bacteria and other microorganisms (Abd Elhameed et al., 2020) that can produce highly active cellulases isolated from soil, decaying branches and leaves, animal intestines, and other stuff (Alonso-Pernas et al., 2017; Tian et al., 2017; Ayumi et al., 2018; Wang et al., 2022).

*Bacillus velezensis*, an endospore-forming gram-positive bacterium belonging to the phylum Firmicutes, is widespread in waterway dregs, soils, and plants, indicating its high ecological adaptability (Balderas-Ruíz et al., 2020; Xu et al., 2020). To date, *B. velezensis* has been widely studied for its ability to efficiently express hydrolases, antibacterial proteins, lipopeptides, and plant hormones, to promote plant growth, and to inhibit plant diseases caused by bacteria and fungi. It has also been reported that *B. velezensis* has a good ability to produce cellulase (Pereira et al., 2019; Lu et al., 2020; Shin et al., 2021; Song et al., 2022). High-throughput sequencing technology is an effective method to analyze the whole genome of *B. velezensis* and mine its related functional genes is an effective method for studying the characteristics of the strain, clarifying its enzyme activity characteristics for degrading lignocellulose, and further increasing the application value of the strain in biomass transformation.

## 2. Materials and methods

### 2.1. Sampling, screening, and detection of strains

Humic soil samples were collected from Qingshuihe County (E 111° 0.68′, N 39° 0.92′), Hohhot City, Inner Mongolia Autonomous Region, China. We weighed 5 g of soil into 45 mL of sterile water and diluted it to different concentration of 10^−1^ - 10^−9^ g/mL. Then, 200 μL of 10^−7^ to 10^−9^ dilutions was applied to carboxymethylcellulose sodium culture (CMC) agar medium (K_2_HPO_4_ 2.5 g/L, Na_2_HPO_4_ 2.5 g/L, peptone 2 g/L, yeast extract 0.5 g/L, carboxymethylcellulose sodium 20 g/L, agar 20 g/L), cultured at 37°C for 24 h. According to the method in Teather and Wood (1982), cellulose degrading bacteria were screened by 0.2% (W/V) Congo red dye. Meanwhile, the selected strains were stained with 1% (W/V) iodine solution (Anand et al., 2010). The isolated strains were inoculated into microcrystalline cellulose (Avicel) agar medium (Avicel 10 g/L, (NH_4_)_2_SO_4_ 1.4 g/L, K_2_HPO_4_ 2.5 g/L, CaCl•2H_2_O 0.3 g/L, MgSO_4_•7H_2_O 0.3 g/L, peptone 2 g/L, yeast extract 0.5 g/L, agar 20 g/L), CMC agar medium and starch agar medium (beef extract 5 g/L, peptone 10 g/L, NaCl 5 g/L, starch 2 g/L, agar 20 g/L). The hydrolytic capacity ratio (HCR) of each strain was determined and expressed as a transparent circle diameter ratio. The screened colonies were confirmed to be single colonies by multiple purification cultures and microscopic examinations, and they were stored in liquid Luria-Bertani (LB) medium (tryptone 10 g/L, yeast extract 5 g/L, NaCl 10 g/L) with 30% glycerol at −80°C.

The morphology of the strain was observed by scanning electron microscope (SEM). The cultures were placed on sterile glass covers in a petri dish. After gently rinsing with PBS, fixation with electron microscope solution (No: G1102, Servicebio, China) was fixed at room temperature for 2 h. The fixed samples were rinsed three times with 0.1 M phosphate-buffered saline (PBS) at pH 7.4, with each rinse lasting 15 min Dehydration with 50, 70, 80, 90, and 100% ethanol for 15 min, respectively, and each concentration was repeated three times. Finally, the sample is dried in a critical point dryer (K850, Quorum, England), coated with gold by an ion sputtering apparatus (No: MC1000, HITACHI, Japan), and observed under a scanning electron microscope (No: SU8100, HITACHI, Japan).

### 2.2. Molecular identification of bacteria

After the activation of the candidate strains, genomic DNA was extracted according to the instructions of the bacterial whole-genome extraction kit (No: DP302, Tiangen Biochemical Technology Co., Ltd., China). Subsequently, 16S ribosomal ribonucleic acid (rRNA) genes was amplified by polymerase chain reaction (PCR) using sequence-specific primers: 27F (5’-AGAGTTTGATCCTGGCTCA-3′) and 1492R (5’-GGTTACCTTGTTACGACTT-3′) (Dobrzyński et al., 2022) in a thermal cycler (Bole T100, United States). The reaction conditions were as follows: 94°C predenaturation (5 min), followed by 35 denaturation cycles (94°C for 30 s), annealing (55°C for 45 s), and extension (72°C for 2 min), and the final repair extension was set at 72°C for 10 min. PCR products were characterized by 1% agarose gel electrophoresis and quantified using a NanoDrop™ One ultra-micro spectrophotometer (Thermo Scientific, USA). The PCR products were sequenced using the Sanger method. Blastn was used to search 16S rRNA gene fragment sequences in the National Center for Biotechnology Information (NCBI) nucleotide database to determine their closest taxonomic relatives (Dashtban et al., 2010). The sequences were used to construct a phylogenetic tree along with other reference genes obtained from NCBI GenBank. A phylogenetic tree was constructed using the MEGA X software neighbor-joining method (1000 bootstrap replications; Kumar et al., 2018).

### 2.3. Cellulase activity assay

Before being placed in liquid CMC medium for culturing, the isolated strains were cultured in liquid LB medium for 18 h. Endocellulase, exocellulase, and glucanase tests were conducted using sodium carboxymethyl cellulose, microcrystalline cellulose, and salicin solutions, and the total cellulase activity was determined using the FPA (Wang et al., 2020). The dinitrosalicylic acid method was used to estimate the reducing sugars released during hydrolysis (Mansour et al., 2016; Yadav and Dubey, 2018). One unit (U) of enzyme activity was defined as the amount of enzyme required to release 1 μmol of reducing sugars per milliliter per minute. The Michaelis–Menten equation was used to calculate the kinetic parameters of the enzymatic reaction of cellulose from strain SSF6. The cellulase activity was calculated using the following formula:


$$\mathrm{Enzyme Activity}\left(U/\mathrm{mL}\right)=\frac{\mathrm{Glucose production}\;\left(\mathrm{mg}\right)\times 1000}{\mathrm{Enzyme dosage}\;\left(\mathrm{mL}\right)\times \mathrm{time}\;\left(\min\right)}$$

### 2.4. Genome sequencing, species assignment and annotation

The extracted genomic DNA was entrusted to Beijing Novogene Bioinformatics Technology Co., Ltd. using an Illumina PE150 system and PacBio high-throughput sequencing technology. Genome assembly was performed using the SMRT Link (version 5.0.1).^1^ The initially assembled data were subjected to low-quality read filtering (less than 500 bp), error correction (selection of long read sequences over 6,000 bp), correction (minimum mass value filtering result 20, minimum read depth 4, maximum read depth 1,000), and cyclization starting checkpoint correction to obtain the final completed map sequence (Ardui et al., 2018; Reiner et al., 2018). GeneMarkS software (version 4.17)^2^ was used for coding gene prediction and filtering (Besemer et al., 2001). Repeat Masker software (version 4.0.5) (Saha et al., 2008) was used for scattered repetitive sequence prediction, and the TRF (Tandem Repeats Finder, version 4.07b) (Benson, 1999) was used to search for tandem repeats in DNA sequences. Transfer RNA (tRNA) genes were predicted using tRNAscan-SE (Lowe and Eddy, 1997). Ribosomal RNA (rRNA) genes were analyzed using the rRNAmmer software (version 1.2) (Lagesen et al., 2007), and small nuclear RNAs (sRNAs) were predicted using the Rfam database software (Nawrocki et al., 2015). The PhiSpy tool (version 2.3) (Zhou et al., 2011) was used to predict prophages.

The genome sequence of *Bacillus* was queried and downloaded from the GenBank genome database.^3^ Typing of the SSF6 genome assembly was determined by calculating Average nucleotide identity (ANI) values through the NCBI Prokaryotic Genome Annotation Pipeline (PGAP) (Tatusova et al., 2016). Meanwhile, orthologous ANI (OrthoANI) was calculated using the orthologous Average Nucleotide Identity Tool (version 1.40) (Lee et al., 2016). The digital DNA–DNA hybridization (dDDH) analysis was carried out using the Genome-to-Genome Distance Calculator (GGDC version 2.1) (Li et al., 2002). BLAST was used to compare the identified genes to the commonly used databases of the NR (NonRedundant Protein), KEGG (Kanehisa et al., 2004, 2006), and the COGs of Proteins (Galperin et al., 2015). For gene function annotation, the CAZy was used (Cantarel et al., 2009).

### 2.5. Comparative genomics analysis

The complete genome sequence of the *B. velezensis* FZB42 (NC_009725) (Fan et al., 2017, 2018) strain was downloaded from the NCBI Genome for comparative genomics analysis with *B. velezensis* SSF6. MUMmer alignment software (version 3.23) was used to detect individual SNPs (Kurtz et al., 2004), and SNP functions were annotated according to positional relationships and interactions between SNPs and genes. Protein sequences of multiple samples to be analyzed were clustered using CD-hit software (version 4.6.1) and plotted using R software (version 3.2.4).

### 2.6. Statistical analysis

GraphPad Prism 9 was used to make glucose standard curves and characteristic enzyme curves. SPSS 20.0 software (IBM SPSS, Chicago, IL) was used for one-way ANOVA analysis of variance for statistical analysis. The data are expressed as the mean ± standard deviation, and a *p* < 0.05 was considered statistically significant.

## 3. Results

### 3.1. Isolation of cellulose-degrading bacteria

**Figure 1:**
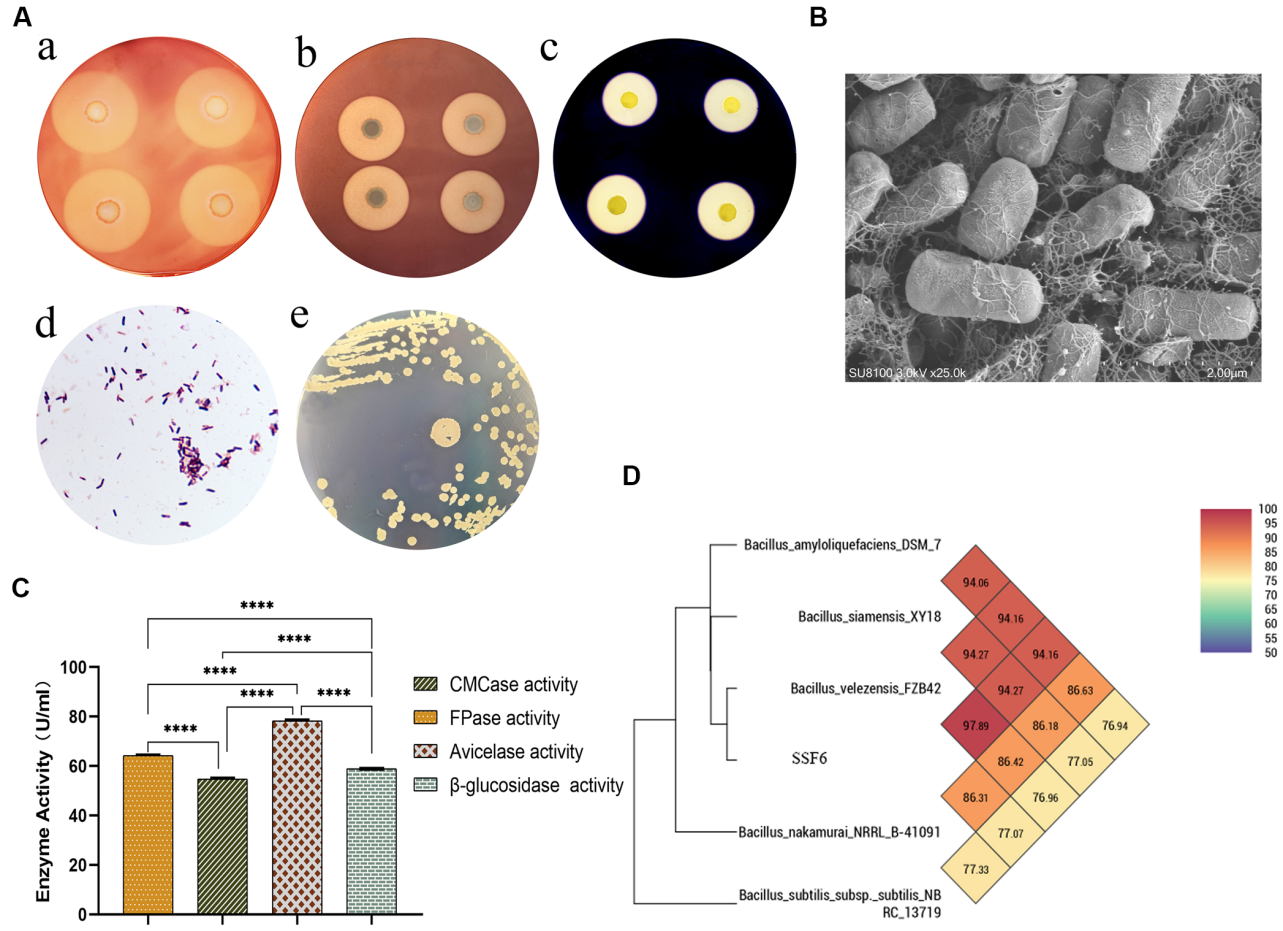
Isolation and Identification of Cellulose Degrading Bacteria. **(A)** Species identification of strain SSF6: **(a)** Cellulose-Degrading Active Regions of Strain SSF6 on CMC Medium; **(b)** Cellulose-Degrading Active Regions of Strain SSF6 on Avicel Medium; **(c)** Cellulose-Degrading Active Regions of Strain SSF6 on Starch Medium; **(d)** Gram staining of strain SSF6; **(e)** Colony morphology of strain SSF6 on LB agar medium. **(B)** Scanning electron microscopy (SEM) observation of strain morphology. **(C)** Characteristics of cellulase activity produced by strain SSF6. **(D)** OrthoANI values were calculated using the genomic sequences of strain SSF6 with other *Bacillus* SPP.

Four strains with good cellulose degradation function, SSF1, SSF4, SSF6, and SSF15, were screened from soil using a CMC selective medium. These four isolated strains, when stained with Congo red solution, produced clear hydrolysis circles around the colonies, the hydrolysis diameters are 4.82 ± 0.13, 7.92 ± 0.16, 26.20 ± 0.44, and 11.56 ± 0.45 mm, respectively, indicating that they have cellulose hydrolysis ability (Table 1). Cellulose-degrading bacteria were screened based on the HCR ratio (Kang et al., 2022), and the hydrolysis ratios of the four isolated strains in the two media were compared. The hydrolysis ratios of each strain were 1.50 ± 0.07, 1.36 ± 0.11, 3.61 ± 0.05, and 3.57 ± 0.32, respectively. Comprehensive analysis revealed that the strain SSF6 had a higher cellulose hydrolysis ratio (Table 2; Figures 1Aa,b) and exhibited excellent cellulose degradation ability. At the same time, we found that the strain SSF6 also had a strong starch degradation ability (3.20 ± 0.12; Table 3; Figures 1Ac), while the other three strains could not degrade starch (Table 3). After comprehensive consideration, strain SSF6 was selected for subsequent experiment and analysis.

**Table 1:** HCR determination for the diameter of the degradation circle of the isolated strain on CMC medium.

Strain	Clearing zone diameter (D, mm)	Colony diameter (d, mm)	HCR (D/d value)
SSF1	4.82 ± 0.13	3.71 ± 0.15	1.30 ± 0.07^c^
SSF4	7.92 ± 0.16	6.48 ± 0.05	1.22 ± 0.03^d^
SSF6	26.20 ± 0.44	6.06 ± 0.08	4.32 ± 0.16^a^
SSF15	11.56 ± 0.45	5.49 ± 0.12	2.11 ± 0.10^b^

Different lowercase letters mean the significance at *p* < 0.05. D, The diameter of the hydrolysis circle; d, The diameter of the colony; HCR (D/d value), The hydrolysis ratio of the strain.

**Table 2:** HCR determination for the diameter of the degradation circle of the isolated strain on Avicel medium.

Strain	Clearing zone diameter (D, mm)	Colony diameter (d, mm)	HCR (D/d value)
SSF1	7.48 ± 0.21	4.99 ± 0.13	1.50 ± 0.07^b^
SSF4	3.16 ± 0.15	2.33 ± 0.16	1.36 ± 0.11^b^
SSF6	22.82 ± 0.36	6.32 ± 0.15	3.61 ± 0.05^a^
SSF15	10.87 ± 0.81	3.05 ± 0.08	3.57 ± 0.32^a^

Different lowercase letters mean the significance at *p* < 0.05. D, The diameter of the hydrolysis circle; d, The diameter of the colony; HCR (D/d value), The hydrolysis ratio of the strain.

**Table 3:** HCR determination for the diameter of the degradation circle of the isolated strain on Avicel medium.

Strain	Clearing zone diameter (D, mm)	Colony diameter (d mm)	HCR (D/d value)
SSF1	-	-	-
SSF4	-	-	-
SSF6	21.37 ± 0.32	6.70 ± 0.16	3.20 ± 0.12
SSF15	-	-	-

Different lowercase letters mean the significance at *p* < 0.05. D, The diameter of the hydrolysis circle; d, The diameter of the colony; HCR (D/d value), The hydrolysis ratio of the strain.

### 3.2. Identification of strain SSF6

The results of morphological identification demonstrated that the SSF6 colony had a smooth and gray surface surrounded by wrinkles, aerobic growth, positive Gram staining, and microscopic rod-shaped bacteria with spores (Figures 1Ad,e, B). Physiological and biochemical analyzes of strain SSF6 were performed using the Biolog GEN III MicroStation automated microbial identification system. There were 24 positive reactions in the carbon source utilization test, including the ability to use cellobiose, sucrose, and fructose as substrates (Supplementary Table S1). Strain SSF6 displayed sensitivity to L-alanine, L-aspartate, L-glutamic acid, and D-aspartate substrates (Supplementary Table S2) and sodium butyrate, sodium sulfite, lithium chloride, and sodium lactate (Supplementary Table S3). Amplified 16S rRNA fragment from strain SSF6 genomic DNA, a 1,500 bp fragment was obtained and submitted to NCBI and performed sequence blast analysis to construct a phylogenetic tree. The results showed that strain SSF6 was closely related to *B. amyloliquefaciens* strain BV2007 (MT613661.1), *B. velezensis* strain 2630 (MT611652.1), and *B. velezensis* strain FZB42 (ON041103.1; Supplementary Figure S1). Therefore, strain SSF6 was identified as *Bacillus* sp.

### 3.3. Determination of cellulase activity in the isolated strains

To further confirm the cellulose degradation ability of bacterial SSF6, the cellulase activity of the strain in CMC medium was determined, including filter paper activity (FPA), endoglucanase, exoglucanase, and β-glucosidase activities. The results illustrated that the filter paper cellulase activity of *B. velezensis* SSF6 was 64.48 ± 0.28 U/mL, endoglucanase activity was 54.39 ± 0.46 U/mL, exoglucanase activity was 78.59 ± 0.42 U/mL, and β-glucosidase activity was 58.96 ± 0.05 U/mL (Figure 1C). The results of the enzymatic reaction demonstrated that the rate of enzymatic reaction was influenced by the substrate concentration. Exoglucanase was most obviously impacted by the microcrystalline cellulose concentration, and it had the highest reaction rate when the substrate concentration was saturated, followed by endoglucanase and β-glucosidase (Supplementary Table S4).

### 3.4. Characterization of the whole genome of strain SSF6

**Figure 2:**
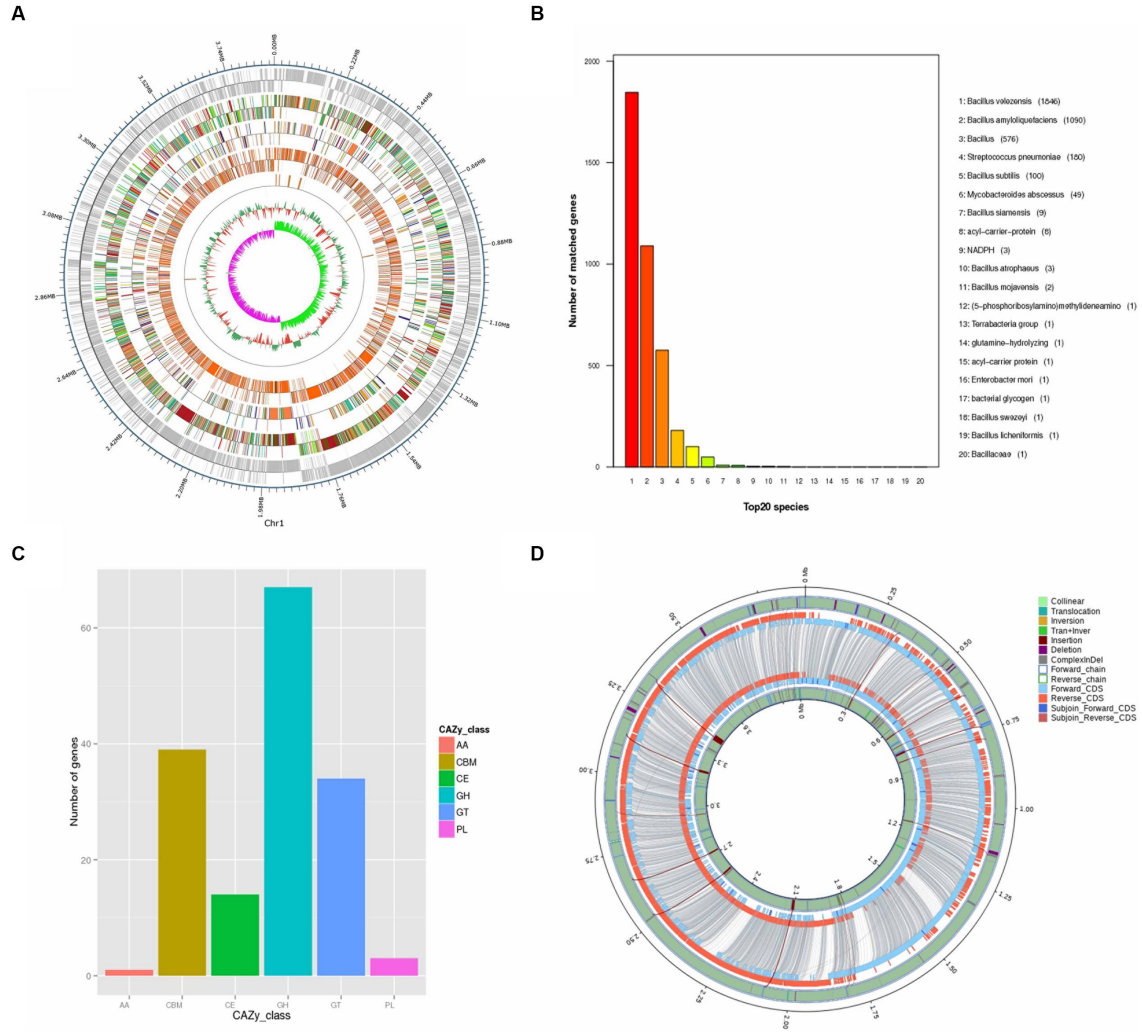
Genomic Analysis of *B. velezensis* SSF6. **(A)** Whole genome completion map. The outermost circle is the position coordinates of the genome sequence, from the outside to the inside, which are the coding genes, gene function annotation results [according to the actual project situation, it may include the annotation result information of COG (KOG), KEGG, GO database], ncRNA, genome GC content: Use window (chromosome length/1000) bp, step size (chromosome length/1000) bp to count GC content, the inward red part indicates that the GC content in this region is lower than the average GC content of the whole genome, and the outward green part is the opposite, and the higher the peak indicates the greater the difference from the average GC content, genome GC skew value: window (staining) Body length/1000 bp, step size (chromosome length/1000) bp, the specific algorithm is G-C/G + C, the inward pink part indicates that the content of G in this region is lower than that of C. The outward light green part is the opposite. **(B)** Non-redundant (NR) protein database annotation. **(C)** Annotated functional classification map of the genome CAZy of *B. velezensis* SSF6. Above is the individual sample ID, the horizontal coordinate is the CAZy database classification type, and the vertical coordinate is the number of genes on the annotation. **(D)** Cycle diagram of structural variation. The inner circle is the sample genome and the outer circle is the reference genome. Collinear: homologous region; Translocation: translocation region; Inversion: inverted region; Tran + Inver: translocation and inverted region; Insertion: insertion region with a length greater than or equal to 50 bp; Deletion: deletion region with a length greater than or equal to 50 bp; ComplexInDel: regions that cannot be aligned but correspond to the position; Forward chain: the forward chain of the genome sequence, the gene coordinates increase clockwise at this time; Reverse chain: the reverse chain of the genome sequence, the gene coordinates increase counterclockwise at this time; Forward CDS: CDS translated on the forward chain of the genome sequence; Reverse CDS: CDS translated on the reverse chain of the genome sequence; Subjoin Forward CDS: Supplementary genome sequence on the forward chain Translated CDS; Subjoin Reverse CDS: CDS translated on the backlink of the complementary genomic sequence, with the paired sequence of DeleteGene or InsertGene as the complementary CDS.

Bacterial lignocellulolytic activity can be better understood using genomic information. In this study, the genome of strain SSF6 was sequenced and the functional genes involved in lignocellulosic degradation were analyzed. Strain SSF6 was assembled into a circular genome after steps of assembly, correction and optimization (Figure 2A). Strain SSF6 had a genome size of 3,891,780 bp, contained 46.67% GC content, three contigs, and N50 contig length 3,893,584 bp (Supplementary Figure S2).

Through genomic analysis of the strain, 4,015 genes with a combined size of 3,499,518 bp were predicted in strain SSF6. The total number of repeats was 380,205 of which were scattered repeats (132 LTR, 19 DNA, 38 LINE, 13 SINE, 2 RC, and 1 unknown), and 175 were tandem repeats. The noncoding RNAs included 86 tRNAs, 27 rRNAs (9 16S rRNAs, 9 5S rRNAs, and 9 23S rRNAs), and 10 sRNAs. Eighteen prophages were predicted, with a total fragment length of 628,164 bp.

The ANI (95–96%) and DNA–DNA hybridization value (DDH,70%) calculated based on genomic nucleic acid sequences have become the gold standard for species classification (Choi et al., 2021). According to ANI calculation by PGAP, strain SSF6 was predicted to be *B. velezensis* with high confidence. The results showed that the top 8 were all *B. velezensis*, with ANI values greater than 97% (Supplementary Table S5). At the same time, OrthoANI values were calculated with five genomic sequences, including SSF6, and the results showed that strain SSF6 had the highest value compared with *B. velezensis* FZB42 (97.59%), followed by *B. siamensis* KCTC 13613 (94.34%) and *B. amyloliquefaciens* DSM 7 (94.02%; Figure 1D). Further calculation of OrthoANI values for 100 selected *B. velezensis* genomes showed that all *B. velezensis* genomes had OrthoANI values greater than 97% (Supplementary Table S6). The dDDH values of strain SSF6 and 100 strains *B. velezensis* genome sequences ranged from 96.32 to 99.1%, where the probability of DDH value ≥70% was greater than 90% (Supplementary Table S6). In summary, strain SSF6 was identified as *B. velezensis* and named *B. velezensis* SSF6.

### 3.5. Gene function annotation

According to the NR database, the number of *B. velezensis* genes annotated by *B. velezensis* SSF6 was the highest (1846), followed by *B. amyloliquefaciens* (1090). This further confirmed that the strain SSF6 was *B. velezensis* (Figure 2B). COG database annotation revealed that the most enriched genes were those involved in the transport and metabolism of amino acids (302 genes) and carbohydrates (249 genes; Supplementary Figure S3). A total of 112 COGs were annotated as being involved in carbohydrate metabolism, including COG2814 (predicted arabinose efflux permease AraJ, MFS family), COG0726 (Peptidoglycan/xylan/chitin deacetylase, PgdA/NodB/CDA1 family), COG1349 (DNA-binding transcriptional regulator of sugar metabolism, DeoR/GlpR family), and COG0697 (Permease of the drug/metabolite transporter (DMT) superfamily).

KEGG integrates genomic, chemical, and system function information and can graphically represent many metabolic pathways and the relationship between various pathways to comprehensively elucidate metabolic pathways. A total of 375 genes related to carbohydrate metabolism were annotated in KEGG (Supplementary Figure S4). Amino sugar and nucleotide sugar metabolism (ko00520, 41 genes), pyruvate metabolism (ko00620, 39 genes), glycolysis/gluconeogenesis (ko00010, 36 genes), starch and sucrose metabolism (ko00500, 34 genes), and the pentose phosphate pathway (ko00030, 25 genes) were the dominant energy metabolism pathways that play a key role in cellulose degradation (Supplementary Table S7). These findings suggested that the *B. velezensis* SSF6 genome contained many genes necessary for metabolizing of carbohydrates and other nutrients, indicating that the strain SSF6 had a significant capacity for carbohydrate polysaccharide degradation.

The genome contained 158 CAZyme genes (3.9% of the total number of genes) with five major classifications: glycoside hydrolases (GHs), glycosyl transferases (GTs), polysaccharide lyases (PLs), carbohydrate esterases (CEs), and auxiliary activities (AAs) (Figure 2C). A total of 67 GH genes were annotated in the genome and assigned to 36 GH families, with the GH1, GH23, GH13-3, and GH4 having the most members. Additionally, carbohydrate-binding module (CBM) genes were identified and assigned to 6 CBM families, which enhanced the catalytic activity by targeting enzymes associated with specific cell wall components (Duan et al., 2017). Moreover, 34 glycosyl transferase (GT) genes were assigned to 9 GT families, along with 3 polysaccharide lyase (PL) genes, and 1 auxiliary activity (AA) gene.

### 3.6. Comparative genomic analysis

The results of genome comparison between *B. velezensis* SSF6 and *B. velezensis* FZB42 showed that the number of genes of the former was greater than that of the latter. In addition to providing molecular evidence for phenotypic differences and similarities, the study of the core genome is of great significance for determining functional differences and similarities between the strains. Core genome analyzes of 2 *Bacillus* genomes were performed. The total number of core genes was 3,341. SSF6-specific genes (557) were more abundant than FZB42-specific genes (340). A total of 156 genes were annotated as having unknown functions (Figure 2D). The core genes related to carbohydrates were annotated to 27 GH (46 genes), 6 CE (13 genes), 8 GT (29 genes), 1 AA (1 gene), 6 CBM (32 genes), and 3 PL (3 genes) families. However, no CAZyme-encoding genes were detected in FZB42, such as the GH13-5 and GH43-8 families. There were 16,027 nonsynonymous SNPs in the SSF6 genome, distributed among 24 glycoside hydrolase families, including GH1, GH4, and GH23 (Supplementary Table S8).

### 3.7. Lignocellulose gene analysis

Genes involved in lignocellulose degradation have been detected in the genome of *B. velezensis* SSF6. A total of 26 cellulase genes were annotated in the *B. velezensis* SSF6 genome, including 2 endoglucanase genes, 10 exoglucanase genes, and 14 β-glucosidase genes (Table 4). The proteins encoded by the endoglucanase, exoglucanase, β-glucosidase genes belong to the GH51, GH1, and GH3 and GH4 families. The number of cellulase genes and family species was similar in SSF6 and FZB42, indicating that *B. velezensis* SSF6 has a strong cellulose degradation potential.

**Table 4:** Annotated common genes encoding lignocellulose degrading enzymes of *B. velezensis* SSF6 strains.

Classifcation	CAZy	Count	Predicted function	EC numbers
Cellulose-related	GH1	8	beta-glucosidase	EC 3.2.1.21
	GH1	8	6-phospho-beta-glucosidase	EC 3.2.1.86
	GH1	8	6-phospho-beta-galactosidase	EC 3.2.1.85
	GH3	2	beta-glucosidase	EC 3.2.1.21
	GH4	4	6-phospho-beta-glucosidase	EC 3.2.1.86
	GH4	4	α-glucosidase	EC 3.2.1.20
	GH5	1	endo-1,4-β-glucanase	EC 3.2.1.4
	GH32	3	endo-levanase	EC 3.2.1.65
	GH51	1	endoglucanase	EC 3.2.1.4
Hemicellulose-related	GH1	8	beta-mannosidase	EC 3.2.1.25
	GH1	8	beta-glycosidase	EC 3.2.1.-
	GH1	8	beta-xylosidase	EC 3.2.1.37
	GH3	2	beta-xylosidase	EC 3.2.1.37
	GH3	2	alpha-L-arabinofuranosidase	EC 3.2.1.55
	GH26	1	beta-mannanase	EC 3.2.1.78
	GH51	2	beta-xylosidase	EC 3.2.1.37
	GH51	2	alpha-L-arabinofuranosidase	EC 3.2.1.55
	GH53	1	endo-beta-1,4-galactanase	EC 3.2.1.89
	CE4	8	acetyl xylan esterase	EC 3.2.1.72
	CE7	1	acetyl xylan esterase	EC 3.2.1.72

*Bacillus velezensis* SSF6 contained 28 genes encoding hemicellulases, including 12 β-xylosidase genes, 9 xylanase genes, and 7 additional enzyme genes (mannosidase, α-L-arabinofuranosidase, and polyarabinose exonuclease genes; Table 4). The β-Xylosidases primarily belong to the GH1, GH3, and GH51 families, the xylanases to the CE4 and CE7 families, and the mannosidases, α-L-arabinofuranosidases, and polyarabinose exonucleases to the GH1, GH3, and GH53 families (Table 4).

## 4. Discussion

Recently, a range of microbes capable of degrading lignin, hemicellulose, and cellulose have been identified in microecological settings, such as soil, compost, anaerobic sludge, and plants, including *Serratia marcescens* (Tang et al., 2022), *B. velezensis* (Li Y. et al., 2020), *Paenibacillus* (Yadav and Dubey, 2018), *Cellulomonas*, *Cytophaga* (Tan et al., 2022) and other microorganisms. Based on different cellulose degradation mechanisms, these microorganisms produce a variety of cellulase with industrial value, resulting in huge economic value. The examination of *B. velezensis* focused primarily on the improvement in enzyme production conditions. At present, the related research of *B. velezensis* mainly focuses on the screening of strains, the optimization of fermentation conditions (Nair et al., 2018; Li F. et al., 2020; Djelid et al., 2022), and the prediction of lignocellulosic degradation function of strains by high-throughput sequencing technology and the mining of corresponding genes (Chen et al., 2018; Tang et al., 2021). As a member of a class of microorganisms with cellulose degradation potential, the *B. velezensis* genome contains abundant carbohydrase genes. They can secrete various cellulases. In this study, genome-wide and comparative genomic analyzes were performed for *B. velezensis* SSF6 to uncover functional genes involved in cellulose degradation. This study will benefit the mining of cellulose-degrading enzyme resources and the development and utilization of *B. velezensis* strains.

*Bacillus velezensis* SSF6 had a total cellulose enzyme activity of 64.48 ± 0.28 U/mL, which was higher than that of *B. velezensis* M2 (33.03 U/mL) (Li F. et al., 2020) and *B. licheniformis* KY962963 (6.19 IU/mL) (Shah and Mishra, 2020). Endoglucanase activity was 54.39 ± 0.46 U/mL, exoglucanase activity was 78.59 ± 0.42 U/mL, and β-glucosidase activity was 58.96 ± 0.05 U/mL, which was higher than that of *B. stratosphericus* BHUJPV-H5 (0.35, 0.02, and 1.33 U/mL, respectively), *B. subtilis* BHUJPV-H12 (0.21, 0.03, and 1.24 U/mL, respectively), *B. subtilis* BHUJPV-H19 (0.23, 0.01, and 2.55 U/mL, respectively), and *B. subtilis* BHUJPV-H23 (0.26, 0.02, and 1.87 U/mL, respectively) (Singh et al., 2019). Normally, cellulose is exploited by the synergistic action of three enzymes, endoglucanase, exoglucanase, and β-glucosidase, which hydrolyze cellulose to glucose monomers (Horn et al., 2012). Comparative analysis revealed that *B. velezensis* SSF6 has a strong comprehensive cellulose degradation ability and may be widely used in agriculture in the future. In the future, enzyme production conditions can be further improved and large-scale cellulase production can be achieved through biotechnology, thereby creating significant economic value (Chen et al., 2018; Zhang et al., 2018).

Genomics can provide information about the functional potential of microorganisms. Similar to *B. velezensis* FZB42, the genome size of *B. velezensis* SSF6 was estimated to be 3,891,780 bp with a 46.67% GC content. Through various database annotations, a significant number of amino acid transport, carbohydrate, and metabolic activities have been anticipated. Additionally, many glycoside hydrolases (GHs) are involved in carbohydrate metabolism (An et al., 2021). It contains various hydrolases that act on glycosidic linkages and can hydrolyse polysaccharide substances, such as cellulose, starch, xylan, and mannose (Du et al., 2021). The GH13 family has 36 members, including α-amylase (EC 3.2.1.1) (Janeček et al., 2014), cyclodextrin glucosyltransferase (EC 2.4.1.19), and α-glucosidase (EC 3.2.1.20) (Zhou et al., 2015), and can hydrolyse starch. Both GH1 and GH4 have the potential for cellulose degradation and can effectively degrade lignocellulosic biomass. GH43 is an important component of xylan degradation and is related to hemicellulose degradation.

In conclusion, these carbohydrases catalyze carbohydrate degradation, modification, and biosynthesis and have many applications. The CBM family enhances the catalytic activity by targeting enzymes linked to specific cell wall components (Duan et al., 2017). The CBM5 module improves the affinity of enzymes, such as endoglucanase, chitinase, and lytic polysaccharide monooxygenase (LPMO), for crystalline cellulose and chitin, improving their efficiency in binding to substrates over a wider pH range (Manjeet et al., 2019). In Bcl PMO10A, CBM5 promotes substrate binding and protects the enzyme from deactivation (Mutahir et al., 2018). CE7 contains acetyl xylan esterase and cephalosporin C deacetylase, which are important catalytic enzymes for synthesizing cephalosporin antibiotics (Vincent et al., 2003). CE4 and CE7 promote xylan dissolution and are involved in hemicellulose degradation. The AA10 family’s LPMOs catalyze the oxidative degradation of crystalline polysaccharides, such as cellulose and chitin. Additionally, they act on the xylan, mannan, and cellulose structures of lignocellulosic biomass to provide more binding checkpoints for glycoside hydrolases, thereby enhancing the accessibility of cellulases to substrates and promoting substrate degradation (Pierce et al., 2017).

Several researchers have highlighted the lignocellulose degradation capabilities of *B. velezensis* and predicted cellulase interactions by elucidating the related capabilities of carbohydrases in the genome (Chen et al., 2022a). Furthermore, abundant cellulases and xylanases increase the release of monosaccharides during straw saccharification through synergistic effects (Zeng et al., 2021), which promotes the conversion of cellulose and hemicellulose into soluble sugar. The genome of *B. velezensis* SSF6 used in this study was rich in cellulase and hemicellulase genes. Compared with *B. velezensis* FZB42, strain SSF6 had more GT4 and GH28 genes. GT4 had the most genes among the GT families. These enzymes utilize not only nucleotide sugar donors but also simple phosphosaccharide and lipid phosphosaccharide donors and have potential therapeutic implications. The GH28 and CE8 families are classified as pectinases and play important roles in pectin degradation. These results indicated that *B. velezensis* SSF6 can as the potential to degrade lignocellulose. However, some genes still have not been annotated, and it is still unclear whether the anticipated gene-phenotype can obtain through expression of these genes, so further investigation and inquiry are still required in future scientific research efforts.

## 5. Conclusion

Owing to its potential to offer additional value, such as sustainable energy from biomass and leftover agricultural resources, cellulose conversion is a topic of interest in biotechnology. The results of the present study showed that *B. velezensis* strain SSF6 has an efficient capacity for cellulose degradation. Whole genome sequencing and comparative genomics analysis of *B. velezensis* SSF6 revealed that it contained a large number of genes from different glycosyl hydrolase (GH) families that are essential for cellulose and hemicellulose biodegradation, indicating that strain SSF6 has broad application prospects for industrial enzyme production in the future. In this study, the genetic basis of lignocellulosic degradation was revealed through genome sequencing and analysis, which provided a new microbial resource for lignocellulosic degradation.

## Data availability statement

The datasets presented in this study can be found in NCBI public database. The accession number for genome sequence of *Bacillus velezensis* strain SSF6 is PRJNA934860. The 16S rRNA gene sequence of *Bacillus velezensis* SSF6 strain is OR229485.

## Author contributions

YG and YL: sample collection and strain culture. JM, CC, and LY: experimental study. TZ and SW: research supervision, data analytics, and manuscript writing. YC and YR: critical revision of a manuscript. SS: design of research ideas and key modifications to manuscripts. ZL and XZ: research sponsors and key changes to manuscripts. All authors contributed to the article and approved the submitted version.

## Funding

This work was supported by the Natural Science Foundation of Inner Mongolia Autonomous Region (2022ZD13); High-end talent training projects of Grassland Talents in Inner Mongolia Autonomous Region; National Natural Science Foundation of China (31860356 and 32260457); The Leading Talent Project of “Science and Technology Leading Talent Team Project of Inner Mongolia Autonomous Region” (2022LJRC0010); Inner Mongolia Agricultural and Animal Husbandry Science and Technology Innovation Fund (2022CXJJN08 and 2022CXJJM04); Ordos City science and technology planning project (2022YY033); Project of National Demonstration base for attracting talents and talents (J2018401).

## Conflict of interest

The authors declare that the research was conducted in the absence of any commercial or financial relationships that could be construed as a potential conflict of interest.

## Publisher’s note

All claims expressed in this article are solely those of the authors and do not necessarily represent those of their affiliated organizations, or those of the publisher, the editors and the reviewers. Any product that may be evaluated in this article, or claim that may be made by its manufacturer, is not guaranteed or endorsed by the publisher.

## Footnotes

^1^https://www.pacb.com/support/software-downloads/

^2^http://topaz.gatech.edu/

^3^https://www.ncbi.nlm.nih.gov/genome/
