# Supplementary material for: Screening and genome-wide analysis of lignocellulose-degrading bacteria from humic soil
Source: Front Microbiol. 2023 Aug 11;14:1167293. doi: 10.3389/fmicb.2023.1167293 (PMC10450921; doi:10.3389/fmicb.2023.1167293)
Supplement: Supplementary file 1 [file Data_Sheet_1.pdf]

Supplementary Material

Screening and Genome-Wide Analysis of Lignocellulose-Degrading Bacteria from Humic Soil

Tianjiao Zhang*, Shuli Wei*, Yajie Liu, Chao Cheng, Jie Ma, Linfang Yue, Yanrong Gao, Yuchen Cheng, Yongfeng Ren

*** Correspondence:** Zhanyuan Lu: lzhy2811@163.com

Xiaoqing Zhao: zhaoxq204@163.com

Shaofeng Su: sushaofeng2020@163.com

Supplementary Table 1. Results of sugar alcohol reaction of isolated strains in GenIII identification plates.

Sugar alcohol types	Reaction results	Sugar alcohol types	Reaction results
D- maltose	-	D-salicin	-
D-trehalose	+	D-mannose	+
D-cellobiose	+	D-fructose	+
Gentiobiose	+	D-galactose	-
sucrose	+	3-formylglucose	-
D-turbiose	-	D-mannitol	+
stachyose	-	D-arabol	-
Raffinose、raffinose	-	glycerin	-
α-D-glucose	-	L-rhamnose	-
melibiose	-	inosine	-
inositol	-	N-acetyl-D-glucose	-
β-formyl-D-glucose	-		

Supplementary Table 2. Results of amino acid reactions of isolates in GenIII identification plates.

Types of amino acids	Reaction results	Types of amino acids	Reaction results
Aminoacetyl-L-proline	-	L-Glutamic acid	+
L-Alanine	+	D-Aspartic acid	+
L-Arginine	-	D-Serine	-
L-Aspartic acid	+	L-Pyroglutamic acid	-
L-Serine	-		

Supplementary Table 3. Antibiotic response results of isolates in GenIII identification plates.

Name of antibiotic	Reaction results	Name of antibiotic	Reaction results
Acetomycin	-	Dimethylamine tetracycline	-
Lincomycin, Sujamycin	-	Tetrazolium Blue	-
Vancomycin	-	Tetrazolium Violet	-
Nalidixic acid	-	Lithium chloride	+
Sodium butyrate Guanidine	+	Sodium bromate	-
hydrochloride HCl	-	Sodium tetradecyl sulfate	-
Sodium tellurite	+	Sodium lactate	+

Supplementary Table 4. Kinetic parameters of cellulose enzymatic reaction in strain SSF6.

Enzyme	Vmax（μmol • g^{-1} • min^{-1}）	Km
Endoglucosidase	18.47	0.0479
Exoglucosidase	241.8	1.418
β- glucosidase	13.71	0.00444

Supplementary Table 5. ANI calculation of the strain SSF6 by PGAP.

Species	Strain	Accession	ANI
Bacillus velezensis	KCTC 13012	GCA_001267695.1	97.7012
Bacillus velezensis	NRRL B-41580	GCA_001461825.1	97.6966
Bacillus velezensis	CBMB205	GCA_002117165.1	97.5891
Bacillus velezensis	CBMB205	GCA_003431885.1	97.5964
Bacillus velezensis	KACC 18228	GCA_001461835.1	97.6506
Bacillus velezensis	FZB42	GCA_000015785.2	97.5978
Bacillus velezensis	KACC 13105	GCA_000960265.2	97.5639
Bacillus velezensis	CBMB205	GCA_900100225.1	97.5731
Bacillus amyloliquefaciens	DSM 7	GCA_000196735.1	94.0289
Bacillus siamensis	XY18	GCA_000966575.1	94.2807
Bacillus siamensis	KCTC 13613	GCA_000262045.1	94.3418
Bacillus nakamurai	NRRL B-41091	GCA_001584325.1	86.6648
Bacillus subtilis	NCTC3610	GCA_900445475.1	81.0082
Bacillus subtilis	NBRC 13719	GCA_006741845.1	81.1405
Bacillus subtilis	6051-HGW	GCA_000344745.1	81.1691
Bacillus subtilis	168	GCA_000789275.1	81.1904
Bacillus subtilis	168	GCA_000009045.1	81.1695
Bacillus subtilis	168	GCA_000155325.1	81.062
Bacillus subtilis	168	GCA_013009385.1	81.1829

Bacillus subtilis	168	GCA_012931705.1	81.162

Supplementary Table 6. List of 100 strains of *B. velezensis* used in OrthoANI and dDDH calculation.

Species	Strain	Accession	OrthoANI	DDH	Model C.I.	Distance	Prob. DDH >= 70%
Bacillus velezensis	CAU_B946	GCA_000283695.1	98.567	94.9	[92.6 - 96.6%]	0.0518	98.65
Bacillus velezensis	M27	GCA_000299615.1	98.6211	96.4	[94.4 - 97.6%]	0.042	98.94
Bacillus velezensis	UCMB5036	GCA_000341875.1	97.8502	96.5	[94.6 - 97.7%]	0.0412	98.96
Bacillus velezensis	AH159-1	GCA_000612565.1	98.6579	93.6	[91 - 95.5%]	0.06	98.36
Bacillus velezensis	SQR9	GCA_000685725.1	97.791	91	[87.9 - 93.4%]	0.0752	97.63
Bacillus velezensis	W2	GCA_000732055.1	97.9025	95.1	[92.8 - 96.7%]	0.0506	98.69
Bacillus velezensis	L-H15	GCA_000833005.1	98.5995	96.4	[94.4 - 97.6%]	0.0421	98.94
Bacillus velezensis	AP183	GCA_000875875.2	97.7471	94.1	[91.6 - 95.9%]	0.0569	98.47
Bacillus velezensis	KACC_13105	GCA_000960265.2	97.7284	96.1	[94.1 - 97.4%]	0.0439	98.89
Bacillus velezensis	L-S60	GCA_000973485.1	98.5891	96.4	[94.5 - 97.7%]	0.0417	98.95
Bacillus velezensis	NJN-6	GCA_000973585.1	98.5713	93.4	[90.7 - 95.3%]	0.0615	98.29
Bacillus velezensis	JJ-D34	GCA_000987825.1	98.5822	92.7	[89.9 - 94.8%]	0.0657	98.12
Bacillus velezensis	NBIF-003	GCA_001440465.1	97.7405	95.3	[93 - 96.8%]	0.0497	98.72
Bacillus velezensis	KACC_18228	GCA_001461835.1	97.9205	95.7	[93.6 - 97.2%]	0.0466	98.81
Bacillus velezensis	FKM10	GCA_001469675.1	97.7778	96.1	[94.2 - 97.5%]	0.0436	98.9
Bacillus velezensis	B25	GCA_001536925.1	98.5667	96.5	[94.7 - 97.8%]	0.0407	98.97
Bacillus velezensis	CC09	GCA_001593395.2	97.7397	91.8	[88.8 - 94%]	0.071	97.86
Bacillus velezensis	AP214	GCA_001647945.1	97.748	93	[90.2 - 95%]	0.064	98.19
Bacillus velezensis	S3-1	GCA_001685645.1	97.7561	96.1	[94.2 - 97.5%]	0.0436	98.9
Bacillus velezensis	LS69	GCA_001687745.1	97.7548	96.1	[94.1 - 97.5%]	0.0437	98.89
Bacillus velezensis	V4	GCA_001692115.1	97.7325	96	[93.9 - 97.3%]	0.0448	98.86

Bacillus velezensis	CFSAN034338	GCA_001709045.1	97.7394	92.2	[89.3 - 94.4%]	0.0686	97.98
Bacillus velezensis	CFSAN034340	GCA_001709115.1	98.6063	92.1	[89.2 - 94.3%]	0.069	97.96
Bacillus velezensis	HJ18-4	GCA_001723375.1	98.5298	91.7	[88.6 - 93.9%]	0.0717	97.82
Bacillus velezensis	M75	GCA_001723585.1	98.6344	94.5	[92.1 - 96.2%]	0.0545	98.56
Bacillus velezensis	KD1	GCA_001752685.2	98.9973	98.1	[96.9 - 98.9%]	0.0278	99.25
Bacillus velezensis	SYBC_H47	GCA_001854345.1	98.7405	95.8	[93.7 - 97.2%]	0.0463	98.82
Bacillus velezensis	GH1-13	GCA_002005345.1	98.8269	93.1	[90.3 - 95.1%]	0.0634	98.22
Bacillus velezensis	sx01604	GCA_002057535.1	97.7334	95.9	[93.8 - 97.3%]	0.0453	98.85
Bacillus velezensis	JTYP2	GCA_002072695.1	97.768	96.1	[94.2 - 97.5%]	0.0436	98.9
Bacillus velezensis	OEE1	GCA_002082365.1	98.618	93.4	[90.8 - 95.4%]	0.0612	98.31
Bacillus velezensis	9D-6	GCA_002105595.1	97.7829	94.4	[91.9 - 96.1%]	0.0555	98.52
Bacillus velezensis	CBMB205	GCA_002117165.1	97.7566	96.1	[94.2 - 97.5%]	0.0436	98.9
Bacillus velezensis	NB91	GCA_002154115.1	98.5756	96.1	[94.1 - 97.5%]	0.0437	98.89
Bacillus velezensis	ZL918	GCA_002157265.1	98.556	95.2	[93 - 96.8%]	0.05	98.71
Bacillus velezensis	GQJK49	GCA_002192235.1	97.7388	96.1	[94.2 - 97.5%]	0.0436	98.9
Bacillus velezensis	NWUMFk_BS10.5	GCA_002204665.1	97.6683	94.8	[92.5 - 96.5%]	0.0525	98.63
Bacillus velezensis	T20E-257	GCA_002205715.1	98.6727	96.5	[94.6 - 97.7%]	0.0413	98.96
Bacillus velezensis	NJAU-Z9	GCA_002238395.1	98.6089	96.6	[94.7 - 97.8%]	0.0406	98.97
Bacillus velezensis	2064018	GCA_002271515.1	98.6201	96.8	[95.1 - 98%]	0.0386	99.02
Bacillus velezensis	2137036	GCA_002272285.1	98.5912	94.8	[92.5 - 96.5%]	0.0525	98.63
Bacillus velezensis	7586-G	GCA_002272465.1	98.4628	92.8	[90 - 94.9%]	0.0651	98.14
Bacillus velezensis	J01	GCA_002290045.1	98.5655	93.2	[90.5 - 95.2%]	0.0628	98.24
Bacillus velezensis	B5	GCA_002747285.1	97.834	95.2	[92.9 - 96.7%]	0.0504	98.7
Bacillus velezensis	FTC01	GCA_002751885.1	98.5801	95.3	[93.1 - 96.8%]	0.0493	98.73
Bacillus velezensis	TJ02	GCA_002764075.1	97.8316	94	[91.5 - 95.8%]	0.0577	98.45

Bacillus velezensis	AGVL-005	GCA_002795885.1	97.5245	88.4	[84.9 - 91.1%]	0.0896	96.66
Bacillus velezensis	PG12	GCA_002835205.1	98.6189	94.9	[92.6 - 96.5%]	0.0521	98.64
Bacillus velezensis	CMT-6	GCA_002845365.1	98.5273	95.6	[93.4 - 97%]	0.0476	98.78
Bacillus velezensis	GFP-2	GCA_002850475.1	98.8189	94.7	[92.3 - 96.3%]	0.0536	98.59
Bacillus velezensis	CGMCC_11640	GCA_002968415.1	97.7067	87.6	[84.1 - 90.4%]	0.0937	96.32
Bacillus velezensis	VCC-2003	GCA_002993245.1	97.7259	95.9	[93.8 - 97.3%]	0.0454	98.85
Bacillus velezensis	DR-08	GCA_003045165.1	97.7301	96.1	[94.2 - 97.5%]	0.0436	98.9
Bacillus velezensis	J7-1	GCA_003047005.1	98.6345	96.6	[94.7 - 97.8%]	0.0406	98.97
Bacillus velezensis	44045	GCA_003047025.1	98.6001	96.6	[94.7 - 97.8%]	0.0406	98.97
Bacillus velezensis	131-4	GCA_003047045.1	98.6529	96.6	[94.7 - 97.8%]	0.0406	98.97
Bacillus velezensis	FS001	GCA_003058025.1	97.7469	91.7	[88.8 - 94%]	0.0712	97.85
Bacillus velezensis	LDO2	GCA_003073455.1	97.7578	96.1	[94.1 - 97.5%]	0.0438	98.89
Bacillus velezensis	Hx05	GCA_003150855.2	98.5596	95.6	[93.4 - 97.1%]	0.0474	98.79
Bacillus velezensis	MG33	GCA_003265765.1	97.642	89.5	[86.2 - 92.1%]	0.0837	97.1
Bacillus velezensis	DSYZ	GCA_003285085.1	97.7945	87.8	[84.3 - 90.6%]	0.0925	96.42
Bacillus velezensis	GF423	GCA_003321435.1	97.7753	92.1	[89.1 - 94.3%]	0.0694	97.94
Bacillus velezensis	OSY-GA1	GCA_003441635.1	98.5485	93.7	[91.1 - 95.6%]	0.0594	98.38
Bacillus velezensis	SK007	GCA_003583945.1	97.7243	95.6	[93.5 - 97.1%]	0.0472	98.79
Bacillus velezensis	JT3-1	GCA_003612755.1	97.7834	96.1	[94.2 - 97.5%]	0.0436	98.9
Bacillus velezensis	ZeaDK315 Endobac16	GCA_004135455.2	97.6606	94.8	[92.4 - 96.5%]	0.0527	98.62
Bacillus velezensis	ANSB01E	GCA_004329055.1	97.761	96.1	[94.2 - 97.5%]	0.0436	98.9
Bacillus velezensis	UTB96	GCA_004331895.1	98.6859	96.6	[94.8 - 97.8%]	0.0401	98.98
Bacillus velezensis	BUU_004	GCA_004337575.1	98.5576	93.4	[90.7 - 95.3%]	0.0617	98.29
Bacillus velezensis	LB002	GCA_004337655.1	98.5484	94.1	[91.6 - 95.9%]	0.057	98.47

Bacillus velezensis	YL1	GCA_005636205.1	97.738	96.1	[94.1 - 97.4%]	0.0438	98.89
Bacillus velezensis	Q2B1	GCA_005843905.1	97.7702	96.1	[94.1 - 97.4%]	0.0438	98.89
Bacillus velezensis	WJH	GCA_006350975.1	98.5916	94	[91.5 - 95.8%]	0.0577	98.45
Bacillus velezensis	A35	GCA_006351595.1	98.624	95.9	[93.8 - 97.3%]	0.0453	98.85
Bacillus velezensis	KT1	GCA_006351615.1	97.9207	94.8	[92.4 - 96.4%]	0.0528	98.62
Bacillus velezensis	A25	GCA_006351675.1	98.5623	96.2	[94.2 - 97.5%]	0.0435	98.9
Bacillus velezensis	TK2	GCA_006351695.1	98.5962	93.2	[90.5 - 95.2%]	0.0626	98.25
Bacillus velezensis	WRN014	GCA_006965525.1	98.5531	93.4	[90.7 - 95.4%]	0.0614	98.3
Bacillus velezensis	LG37	GCA_006974185.1	97.757	96.1	[94.2 - 97.5%]	0.0436	98.9
Bacillus velezensis	JK	GCA_007655015.1	97.7487	96.1	[94.1 - 97.5%]	0.0438	98.89
Bacillus velezensis	DE0372	GCA_007673335.1	97.7848	93.3	[90.6 - 95.3%]	0.0621	98.27
Bacillus velezensis	Fad_94	GCA_007845055.1	99.0225	97	[95.3 - 98.1%]	0.037	99.06
Bacillus velezensis	LC1	GCA_008802875.1	97.7436	96.1	[94.2 - 97.5%]	0.0436	98.9
Bacillus velezensis	BPC6	GCA_009193045.1	97.7774	95.1	[92.9 - 96.7%]	0.0505	98.69
Bacillus velezensis	BvL03	GCA_009664145.1	98.9325	96.3	[94.4 - 97.6%]	0.0425	98.92
Bacillus velezensis	VRA_336g_f	GCA_009683055.1	98.5697	94.1	[91.5 - 95.9%]	0.0575	98.45
Bacillus velezensis	VRA_336g_n	GCA_009683125.1	98.5995	94.6	[92.2 - 96.3%]	0.0541	98.58
Bacillus velezensis	VRA_517_n	GCA_009683155.1	98.6199	93.7	[91 - 95.5%]	0.0599	98.36
Bacillus velezensis	GA1	GCA_009734085.1	98.5467	96.6	[94.7 - 97.8%]	0.0406	98.97
Bacillus velezensis	HN-Q-8	GCA_009738165.1	97.7974	95.3	[93 - 96.8%]	0.0496	98.72
Bacillus velezensis	SRCM102742	GCA_009913335.1	97.7827	95.3	[93 - 96.8%]	0.0496	98.72
Bacillus velezensis	SRCM102747	GCA_009913495.1	97.7807	93.6	[91 - 95.5%]	0.0602	98.35
Bacillus velezensis	SRCM101368	GCA_009914175.1	98.6253	93	[90.2 - 95%]	0.064	98.19
Bacillus velezensis	12145	GCA_009935125.1	98.5831	94.2	[91.7 - 96%]	0.0568	98.48
Bacillus velezensis	11749	GCA_009935175.1	98.5745	94	[91.4 - 95.8%]	0.058	98.43

Bacillus velezensis	43859	GCA_009935255.1	97.726	95.3	[93.1 - 96.8%]	0.0494	98.73
Bacillus velezensis	43882	GCA_009935455.1	97.7107	95.3	[93.1 - 96.8%]	0.0495	98.72
Bacillus velezensis	43969	GCA_009935495.1	97.6817	95.2	[93 - 96.8%]	0.0498	98.72
Bacillus velezensis	K1	GCA_009996865.1	97.7999	92.5	[89.6 - 94.6%]	0.0668	98.06
Bacillus velezensis	Marseille-Q1230	GCA_902703385.1	98.7695	97.2	[95.6 - 98.3%]	0.0353	99.1

Supplementary Table 7. KEGG pathways related to carbohydrate metabolism in SSF6 genome.

serial number	ID of path	pathways	number	ratio
1	ko00500	Starch and sucrose metabolism	34	0.91
2	ko00010	Glycolysis / gluconeogenesis	36	0.96
3	ko00620	Pyruvate metabolism	39	1.05
4	ko00030	Pentose phosphate pathway	25	0.67
5	ko00520	Amino and nucleotide sugar metabolism	41	1.10
6	ko00051	Fructose and mannose metabolism	20	0.54
7	ko00650	Butanoate metabolism	23	0.62
8	ko00040	Pentose and glucuronate interconversions	19	0.51
9	ko00640	Propanoate metabolism	31	0.83
10	ko00630	Glyoxylate and dicarboxylate metabolism	34	0.91
11	ko00020	Citrate cycle (TCA cycle)	23	0.62
12	ko00053	Ascorbate and aldarate metabolism	5	0.13
13	ko00052	Galactose metabolism	21	0.56
14	ko00660	C5-Branched dibasic acid metabolism	9	0.24
15	ko00562	Inositol phosphate metabolism	15	0.40

Supplementary Table 8. GH family outcomes with nonsynonymous SNP mutations.

CAZy	Predicted function	EC number
GH1	beta-glucosidase	EC 3.2.1.21
	beta-galactosidase	EC 3.2.1.23
	beta-mannosidase	EC 3.2.1.25
	6-phospho-beta-galactosidase	EC 3.2.1.85
	6-phospho-beta-glucosidase	EC 3.2.1.86
GH3	beta-glucosidase	EC 3.2.1.21
GH4	alpha-glucosidase	EC 3.2.1.20
	6-phospho-beta-glucosidase	EC 3.2.1.86
GH5	endo-beta-1,4-glucanase / cellulase	EC 3.2.1.4
	alpha-glucosidase	EC 3.2.1.20
	glucodextranase	EC 3.2.1.70
GH13	alpha-glucosidase	EC 3.2.1.20
	glucodextranase	EC 3.2.1.70
GH16	endo-1,3(4)-beta-glucanase	EC 3.2.1.6
	beta-glucosidase	EC 3.2.1.21
	exo-beta-1,4-glucanase / cellodextrinase	EC 3.2.1.74
	cellulose beta-1,4-cellobiosidase	EC 3.2.1.91
GH18	endo-beta-N-acetylglucosaminidase	EC 3.2.1.96
GH20	-	-
GH23	peptidoglycan lyase	EC 4.2.2.n1
GH26	beta-mannanase	EC 3.2.1.78
	exo-beta-1,4-mannobiohydrolase beta-1,3-	EC 3.2.1.100
	xylanase	EC 3.2.1.32
GH28	xylogalacturonan hydrolase	EC 3.2.1.-
	Polygalacturonase	EC 3.2.1.15
	exo-polygalacturonase	EC 3.2.1.67
GH32	endo-levanase	EC 3.2.1.65
GH36	alpha-galactosidase	EC 3.2.1.22
	alpha-N-acetylgalactosaminidase	EC 3.2.1.49
GH43	-	-
GH51	endoglucanase	EC 3.2.1.4
GH53	endo-beta-1,4-galactanase	EC 3.2.1.89
GH68	Levansucrase	EC 2.4.1.10
	beta-fructofuranosidase	EC 3.2.1.26
	inulosucrase	EC 2.4.1.9
GH73	Lysozyme	EC 3.2.1.17
	mannosyl-glycoprotein endo-beta-N-acetylglucosaminidase	EC 3.2.1.96
GH100	alkaline and neutral invertase	EC 3.2.1.26
GH101	endo-alpha-N-acetylgalactosaminidase	EC 3.2.1.97

GH126	alpha-amylase	EC 3.2.1.-
GH170	6-phospho-N-acetylmuramidase	EC 3.2.1.-
GH171	peptidoglycan beta-N-acetylmuramidase	EC 3.2.1.92

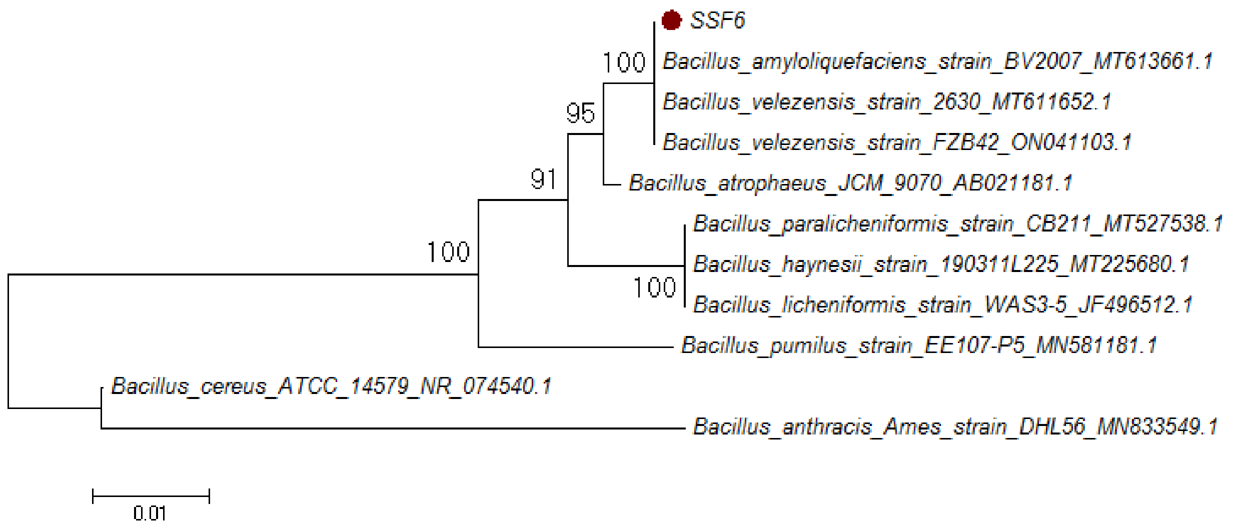

Supplementary Figure 1. Phylogenetic tree derived from 16S rRNA gene sequence.

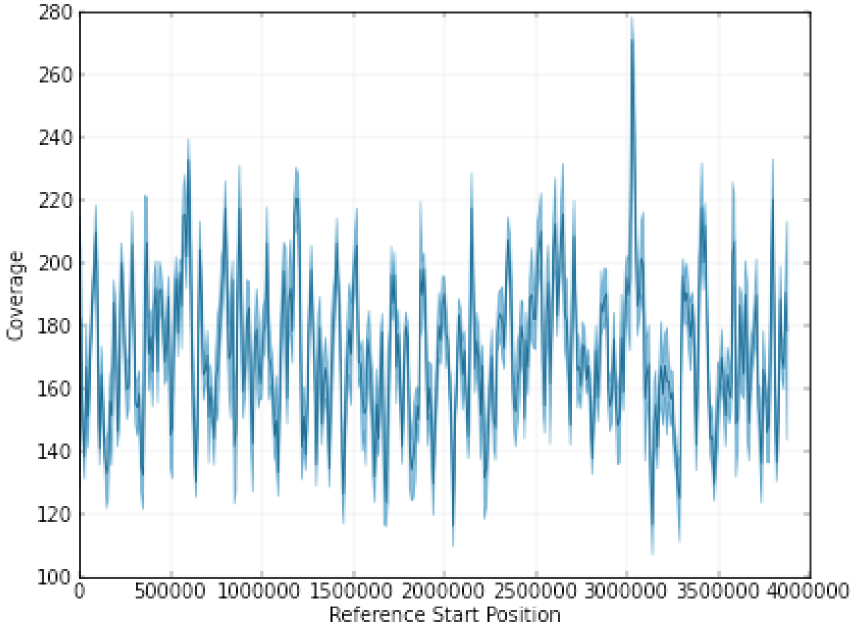

Supplemntary Figure 2. Strain SSF6 assembly results sequencing depth profile.

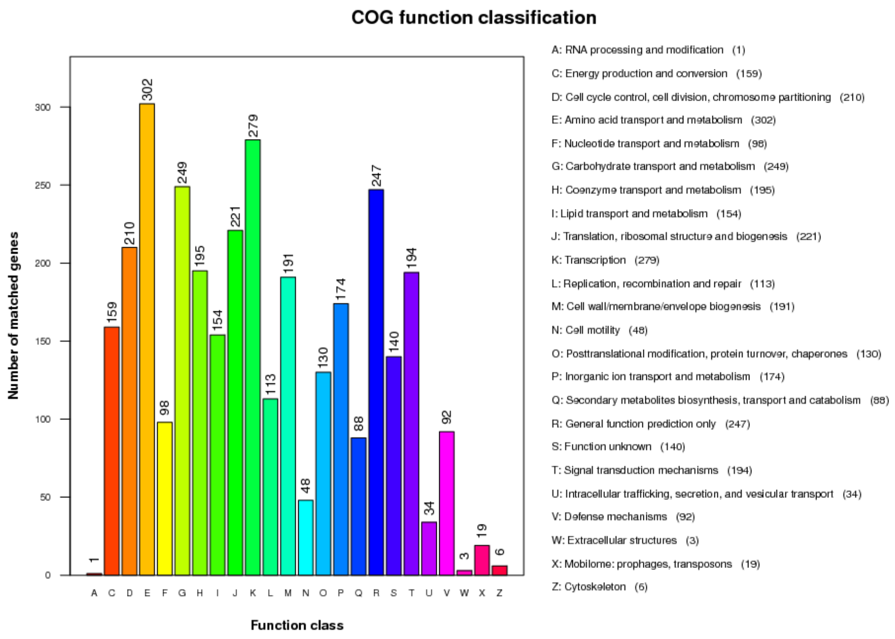

Supplementary Figure 3. Functional annotation of the genome COG of *B. velezensis* SSF6.

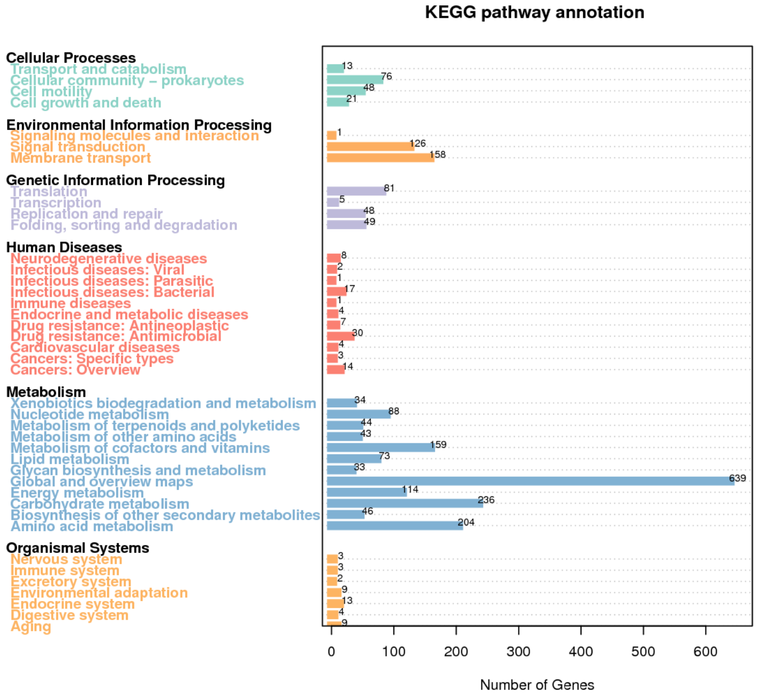

Supplementary Figure 4. KEGG functional annotation of *B. velezensis* SSF6.
